# Antibiotic prescription during the COVID-19 pandemic: A biphasic pattern

**DOI:** 10.1017/ice.2020.381

**Published:** 2020-07-30

**Authors:** Gabriela Abelenda-Alonso, Ariadna Padullés, Alexander Rombauts, Carlota Gudiol, Miquel Pujol, Claudia Alvarez-Pouso, Ramón Jodar, Jordi Carratalà

**Affiliations:** 1 Department of Infectious Diseases, Bellvitge University Hospital, Barcelona, Spain; 2 Bellvitge Biomedical Research Institute (IDIBELL), Barcelona, Spain; 3 Department of Pharmacy, Bellvitge University Hospital, Barcelona, Spain; 4 University of Barcelona, Barcelona, Spain; 5 Spanish Network for Research in Infectious Diseases, Madrid, Spain

*To the Editor—*The COVID-19 pandemic, caused by the severe acute respiratory syndrome coronavirus 2 (SARS-CoV-2), is a public health problem of historic dimensions. However, this pandemic is occurring in the setting of an antimicrobial resistance crisis that is increasing at an alarming pace worldwide. Of concern, countries with a particularly high incidence of COVID-19 also have significant rates of infection caused by multidrug-resistant bacteria. During the 2009 influenza pandemic, coinfection with bacteria was identified as a prognostic factor for the worse outcomes.^1^ This finding has led to empirical antibiotic therapy being recommended for patients with suspected influenza pneumonia,^2^ and it has probably been a major reason underpinning the initial World Health Organization’s recommendation to use empirical antibiotics in cases of COVID-19 pneumonia.^3^ Although this guideline advocated for early antimicrobial de-escalation, a couple of factors may have hindered this practice. First, processing microbiological samples in saturated emergency rooms and overloaded laboratories is difficult. Second, no evidence-based antiviral treatment for COVID-19 has been developed in the setting of a highly stressful situation. Together, these factors may have prompted clinicians to prescribe broad-spectrum antimicrobials more often than they may otherwise have. Therefore, antimicrobial stewardship approaches urgently need to be reinforced during the COVID-19 pandemic.^4^ To date, however, no study has evaluated the impact of the COVID-19 pandemic on antibiotic consumption.

We conducted a before-and-after cross-sectional study comparing data in 2019 (before the COVID-19 pandemic began) and 2020 (COVID-19) for the periods from January 1 to April 30. Bellvitge University Hospital is a 700-bed hospital that serves as a public referral center of 1 million inhabitants in Catalonia, the second worst pandemic-affected area in Spain.^5^ As of April 30, 2020, this hospital had had >1,293 hospital admissions for COVID-19, with a 317% increase in critical care bed use. In this study, we calculated the defined daily dose per 100 patient days, as described elsewhere, and based on the dispensing data of our electronic prescribing system. Medians for continuous variables were compared using the Wilcoxon log-rank test.

**Fig. 1.**
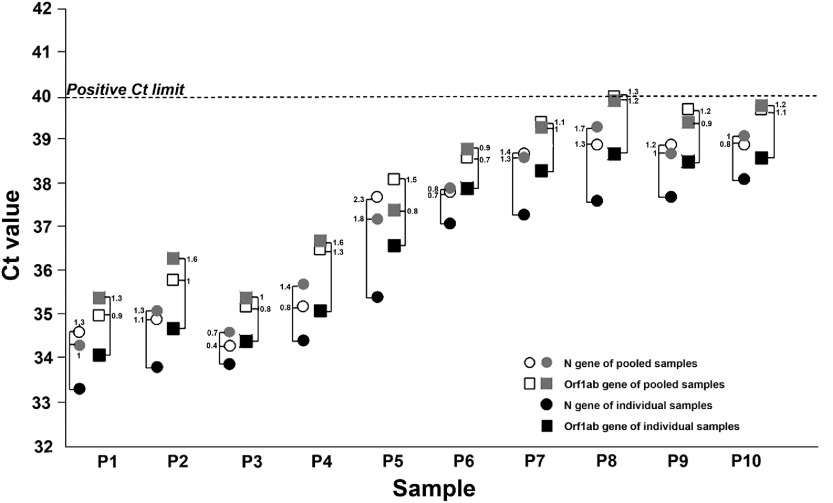
Total antimicrobial consumption and comparative consumption of a amoxicillin/clavulanate and broad-spectrum antibiotics during the first 4 months of 2019 and 2020. Broad-spectrum antibiotics included cefepime, piperacillin/tazobactam, meropenem, imipenem, and ertapenem. Note. DDD, defined daily dose.

Antibiotic use was similar in January and February of both 2019 and 2020, with a slightly lower consumption in 2020 (Fig. 1). As expected, as the COVID-19 pandemic dramatically progressed through March and April 2020, the overall monthly antibiotic usage increased significantly compared with 2019 (*P* < .001). Interestingly, we observed a biphasic phenomenon. During the first peak in March 2020, amoxicillin/clavulanate use trended upward rapidly, consistent with hospital recommendations regarding empirical antibiotic treatment in patients with COVID-19. During the second peak in April 2020, however, we observed a significant increase in broad-spectrum antibiotic prescribing and a slight decrease in amoxicillin/clavulanate use. The biphasic pattern of antibiotic use was associated with 2 specific moments of the pandemic. The first moment corresponded to the empirical coverage of all cases of COVID-19 pneumonia and a high admission rate to our hospital (1,269 patients between March 12 and April 12). The second moment corresponded to a phase in which admission to critical care units accumulated for patients with more severe disease, probably corresponding with an increase in nosocomial infection.

Despite the lack of formal recommendation for the inclusion of antimicrobial stewardship programs in emergency response preparedness efforts, it has been suggested that these programs are essential to mitigate the damage caused by the pandemic.^6^ The initial uncertainty and the difficulty in obtaining microbiological results have been determining factors in the difficulty of implementing an antimicrobial stewardship approach during the COVID-19 pandemic. This lack of information can be explained by several factors: insufficient follow-up times, the heterogeneous use of immunomodulatory drugs, and differences in the availability of infection control measures based on the number of cases per center.^7^ As has been recently shown, bacterial coinfection in COVID-19 appears to be scarce (2.1%).^8^ Notably, superinfection in critically ill patients might be higher (13.5%), and up to 94% of critically ill patients are treated with antibiotics.^9^ Nevertheless, we are not aware of studies that have specifically investigated the development of superinfection by antimicrobial-resistant microorganisms in COVID-19 patients. In this scenario, we think that standardizing immunomodulatory treatments, ensuring compliance with usual infection control practices, and carefully interpreting microbiological results will be key measures that could favor a more cautious approach to the use of antibiotics. We identified a biphasic pattern of increased antibiotic use that corresponded with a first wave of empirical antibiotic therapy and a second biphasic pattern with higher use of broad-spectrum antibiotics. To the best of our knowledge, this is the first description of antibiotic use dynamics during the COVID-19 pandemic. However, this study has some limitations. First, we did not provide information about the clinical indications for the antibiotic use. Second, as a single-center study, local factors that preclude the extrapolation of our findings to other centers.

In conclusion, our data support the World Health Organization concern regarding inappropriate use of antibiotics during the pandemic and the recent change in its guidelines discouraging empirical antibiotics in COVID-19.^10^ Long-term studies are needed to assess the impact of the increase in antibiotic use during COVID-19 pandemic on the hospital flora, and in turn, how this might affect future nosocomial infection and antimicrobial resistance trends worldwide. Meanwhile, it is crucial to standardize the use of antimicrobial stewardship principles to provide the safest therapeutic strategy not only for our present patients but also our future patients.
