# Antibiotic prescription during the COVID-19 pandemic: a biphasic pattern – ERRATUM

**DOI:** 10.1017/ice.2020.426

**Published:** 2020-11

In the above mentioned article by Abelenda-Alonso et al^1^, the wrong figure file was used for Figure 1 in the final published version of record. The correct Figure 1 appears below. The publisher apologizes for the error.

**Fig. 1.**
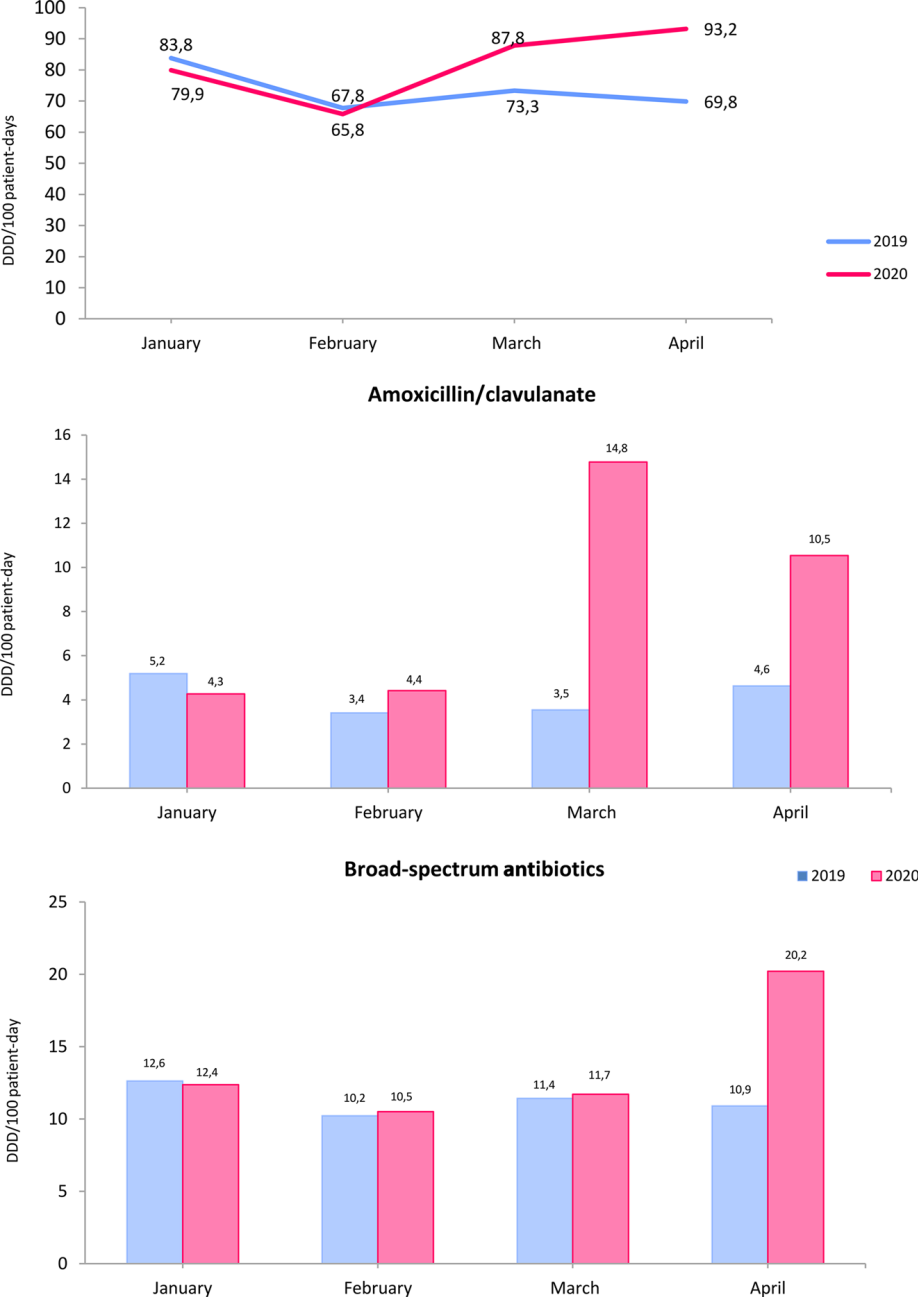
Total antimicrobial consumption and comparative consumption of a amoxicillin/clavulanate and broad-spectrum antibiotics during the first 4 months of 2019 and 2020. Broad-spectrum antibiotics included cefepime, piperacillin/tazobactam, meropenem, imipenem, and ertapenem. Note. DDD, defined daily dose.
